# Inferring muscle functional roles of the ostrich pelvic limb during walking and running using computer optimization

**DOI:** 10.1098/rsif.2016.0035

**Published:** 2016-05

**Authors:** Jeffery W. Rankin, Jonas Rubenson, John R. Hutchinson

**Affiliations:** 1 Structure and Motion Laboratory, Department of Comparative Biomedical Sciences, The Royal Veterinary College, Hawkshead Lane, Hatfield, Herts, UK; 2 Department of Kinesiology, Pennsylvania State University, University Park, PA, USA; 3 School of Sport Science, Exercise and Health, The University of Western Australia, Perth, Western Australia, Australia

**Keywords:** musculoskeletal model, inverse dynamics, forward dynamics, OpenSim, static optimization, computed muscle control

## Abstract

Owing to their cursorial background, ostriches (*Struthio camelus*) walk and run with high metabolic economy, can reach very fast running speeds and quickly execute cutting manoeuvres. These capabilities are believed to be a result of their ability to coordinate muscles to take advantage of specialized passive limb structures. This study aimed to infer the functional roles of ostrich pelvic limb muscles during gait. Existing gait data were combined with a newly developed musculoskeletal model to generate simulations of ostrich walking and running that predict muscle excitations, force and mechanical work. Consistent with previous avian electromyography studies, predicted excitation patterns showed that individual muscles tended to be excited primarily during only stance or swing. Work and force estimates show that ostrich gaits are partially hip-driven with the bi-articular hip–knee muscles driving stance mechanics. Conversely, the knee extensors acted as brakes, absorbing energy. The digital extensors generated large amounts of both negative and positive mechanical work, with increased magnitudes during running, providing further evidence that ostriches make extensive use of tendinous elastic energy storage to improve economy. The simulations also highlight the need to carefully consider non-muscular soft tissues that may play a role in ostrich gait.

## 1. Introduction

Ostriches (*Struthio camelus*) walk and run with high metabolic economy [1–3], can reach very fast running speeds [4,5], and quickly execute cutting (turning) manoeuvres [6]. The ability to achieve such impressive performance is thought to largely arise from morphological specializations within the pelvic limbs as result of their cursorial and secondarily flightless evolutionary background. Like other birds, ostriches use three-dimensional limb joint motions during locomotion [6–8] and have specialized passive structures at the hip, including bony stops (e.g. the antitrochanter), which play an unclear role during movement [9–14]. The distal limb muscles are also highly specialized, consisting of extremely long tendons that cross mobile metatarsophalangeal (MTP) joints. Experimental studies of these features in ostriches and other birds support the inference that they improve gait performance and economy [2,15–18]. However, these adaptations also contribute to the extremely complex ostrich pelvic limb musculoskeletal structure, which consists of more than 30 muscles—the majority of which are multiarticular—that cross joints with multiple degrees of freedom (DOF). As a result, little can be intuitively inferred about specific functional roles that individual pelvic limb muscles perform in ostriches (or many other birds) during gait. Obtaining the data required to determine muscle function is further limited owing to the numerous challenges associated with the required experimental techniques (e.g. electromyography (EMG), sonomicrometry, tendon buckles). To date, these factors have obscured how ostriches and other birds successfully meet the biomechanical demands of walking and running.

During a movement, the functional role of a muscle–tendon unit (MTU) can be established based on a combination of muscular force generation and muscle and tendon length trajectories [19–21]. If an MTU generates high force and positive power (concentric contraction) during the movement, then energy is added to the system and the MTU can be classified as a ‘motor’. In contrast, an MTU that generates high force but negative power (eccentric contraction) removes energy from the system and acts as a ‘brake’. In some cases, an MTU may generate high forces but produce very little positive or negative power (i.e. no length change) during the movement. In this case, the MTU has not added or removed energy from the system and acts as a joint stabilizer or ‘strut’. Last, an MTU may generate high force and switch from negative to positive power production. In this case, the net energy provided to the system is again near zero. However, the MTU has undergone a systematic change in length and likely acts as a ‘spring’, storing energy from an earlier portion of the movement that can be released later. To define an MTU's functional role(s) in this study, muscle excitation timing is first used to classify whether or not a muscle primarily contributes to ‘stance’ (i.e. when the foot is in contact with the ground) or ‘swing’ (i.e. no foot–ground contact) movements, when possible [22,23]. Following this classification, specific muscle roles (i.e. motor, brake, strut or spring) during stance and swing are then determined using MTU force and length. These roles can then be used to infer how individual muscles contribute to the overall mechanical energy flow during gait.

Because the aforementioned difficulties associated with experimental approaches limit their usefulness, an alternative approach is to use realistic, detailed musculoskeletal models and simulations. The first simple ostrich model was developed over 35 years ago by Alexander *et al.* [4] to estimate muscle and bone stress during running. More recently, two-dimensional ostrich models have been developed to investigate postural effects on running joint mechanics [5] and to validate running posture [24] and maximal speed [25] predictions for various extinct taxa. Until very recently, only a single model of locomotion has included muscle geometry, which was limited to six muscles [25]. However, we have just published a highly detailed musculoskeletal model of an ostrich's pelvic limbs, building on prior efforts [26]. Similar approaches have been successfully used to address many questions in human gait: providing insights into muscle function [27–29] and form–function relationships [30,31].

Like most animal musculoskeletal systems, the ostrich pelvic limb has many more muscles than DOF. As a result, multiple muscle excitation patterns exist to produce identical joint mechanics. Knowing how to correctly ‘parse’ the different muscle contributions to the net joint mechanics during movement is critical to understanding muscle functional roles. Two distinct approaches have been used to overcome this major challenge: static and dynamic optimization [32–34]. Static optimization (SO) addresses each instant in time as an independent data point, reducing computational cost but ignoring time-dependent quantities such as activation–deactivation dynamics and tendon strain energy. Dynamic optimization techniques can account for these time-dependent quantities, but incur a high computational cost. There remains considerable debate over which (if either) is more suitable than another for studying muscle function during movement, in large part because a gold standard (i.e. empirical dataset) is not readily available for comparison. For example, Anderson & Pandy [35], after simulating half-gait cycles of human walking, suggested that static and dynamic optimization solutions were ‘practically equivalent’, but qualified their statement and provided scenarios in which dynamic optimization may be necessary. Later comparisons between the two approaches in other human movements have been inconclusive in determining a preferred technique for predicting muscle activity [36–38]. Because of the large number of differences that exist between humans and ostriches in both limb morphology and gait mechanics [2], determining how sensitive muscle functional roles (and by extension structure–function relationships) between these two techniques during ostrich gait could help future comparative research focused on movement in different species.

The primary purpose of this study was to determine the functional roles that individual pelvic limb muscles have in ostriches during walking and running. Existing biomechanical data were combined with a newly developed, detailed ostrich musculoskeletal model [39] to generate computer simulations that estimate MTU excitation, length and force during the two gaits. A secondary purpose was to assess how sensitive muscle functional roles are to choice of optimization approach (static versus dynamic) using a model that widely diverges in morphology from humans and a higher speed movement than those investigated previously. These two purposes are linked, because methodological assumptions of static versus dynamic analysis [5,25,35] might influence biological conclusions about the functions of particular muscles, which can be tested by achieving these two major aims.

## 2. Methods

A detailed musculoskeletal model of the ostrich pelvic limb [39] was combined with experimental data obtained from a representative walking and running trial [2,8,39] within OpenSim [40] to generate six different simulations (three for each motion, table 1). Two simulations (*W*_SO_, *R*_SO_) were performed using OpenSim's SO routine [41]. Two additional simulations (*W*_CMCC_, *R*_CMCC_) were then generated using OpenSim's computed muscle control (CMC) routine [42]. The final two simulations (*W*_CMCR_, *R*_CMCR_) were generated using CMC, but tendons were constrained to be rigid in order to provide a direct comparison with the SO solution, which did not incorporate tendon dynamics, whereas the other two CMC simulations (*W*_CMCC_, *R*_CMCC_) did. The simulations estimated MTU excitation patterns, force and length, which were used to infer muscle function. Details of the musculoskeletal model, optimization framework and experimental data are given below.

**Table 1.** Names and description of the six simulations performed. Simulations were performed for either a walking or running motion (rows) using three different optimization frameworks (columns).

	simulation		
motion	static optimization	computed muscle control (rigid tendon)	computed muscle control (compliant tendon)
walking	*W*_SO_	*W*_CMCR_	*W*_CMCC_
running	*R*_SO_	*R*_CMCR_	*R*_CMCC_

### 2.1. Musculoskeletal model

**Figure 1.**
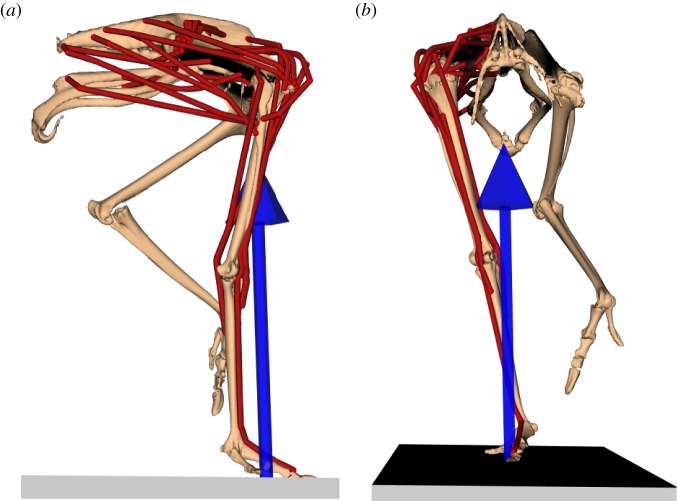
Musculoskeletal model at mid-stance during running. The arrow (blue) indicates the direction and location (centre of pressure) of the ground reaction force. Muscle–tendon actuators (red lines) of the left limb were replaced by idealized joint actuators. (*a*) Sagittal view. (*b*) Frontal view.

The original musculoskeletal model was created using muscle and tendon architecture, digitized muscle paths and computed tomography (CT) scan data collected via dissection [39]. The left pelvic limb was generated by mirroring the right-side segments, joint definitions and muscle tendon paths about the sagittal plane. The model consisted of nine rigid body segments representing the pelvis and left and right-side femur, tibiotarsus, tarsometatarsus and pes (figure 1). The original model's segment mass and inertia values were scaled using the original ostrich's body mass (65.3 kg) and mass of the bird that provided the experimental data (78.7 kg; see §2.3).

Each pelvic limb had 8 DOF representing the hip (3 DOF), knee (3 DOF), ankle (1 DOF) and MTP (1 DOF) joints. In the original model, both the ankle and MTP joints were modelled as 3 DOF (ball-and-socket) joints. However, minimal long axis rotation and ad/abduction have been observed in the avian ankle and MTP during walking and running [7,8,43,44] and these DOFs were constrained to match experimental mid-stance values. The pelvis moved freely relative to the ground (i.e. three translational and three rotational DOFs).

Model segments were driven by a combination of musculotendon and idealized joint (coordinate) actuators (figure 1). Thirty-four of the 35 musculotendon actuators from the original model were retained on the right side, which represented the major muscles in the ostrich pelvic limb (FCLA was removed due to its very low maximum force [39]). Musculotendon actuators were modelled using a Hill-type model that included intrinsic force–length–velocity relationships [45]. Because walking and running are everyday activities and critical to survival, it is likely that MTU properties are tuned so force and power generation are near optimal during these movements [46,47]. However, many muscles in the original model did not reflect this, with normalized fibre lengths exceeding the physiological operating range of 0.5–1.5 optimal fibre lengths in some postures. In the original model, tendon slack lengths (*L*_tsl_) were estimated based on joint range of motion [39,48], which may not reflect tuning for major activities like gait. To correct for this inconsistency, the original model's *L*_tsl_ were systematically adjusted, so that muscle fibre lengths operated over a more optimal range (i.e. 0.75–1.25 optimal fibre length) in the joint ranges of motion defined by the experimental gait kinematics. New *L*_tsl_ were within 10% of the original model values for all actuators except for M. iliotibialis (ILa, ILp, 19%) and M. femorotibialis intermedius (FMTIM, 19%). Maximum isometric forces were scaled using the mass ratio between the original model and experimental subject (table 2). For all musculotendon actuators, maximum contraction velocity was set to 14 *L*_fopt_ s^−1^ [49]. Excitation–activation dynamics were represented by a first-order differential equation with activation and deactivation time constants of 10 and 15 ms. As the left side's movement was assumed to be symmetric with the right side (see §2.3 Experimental data), the model was simplified by having the left side's joints actuated by eight idealized torque actuators—one for each DOF.

**Table 2.** Muscle–tendon actuator properties. Optimal fibre lengths and pennation angles are from the original model by Hutchinson *et al.* [39] but provided for reference.

abbreviation	muscle name	maximum isometric force (*F*_iso_, N)	optimal fibre length (L_fopt_, m)	tendon slack length (*L*_tsl_, m)	pennation angle (°)
IC	M. iliotibialis cranialis	889	0.174	0.0451	0
ILa	M. iliotibialis lateralis (cranial part)	1265	0.174	0.2432	0
ILp	M. iliotibialis lateralis (caudal part)	1265	0.174	0.3099	0
AMB1	M. ambiens, ventral (pubic) head	971	0.039	0.1648	10
AMB2	M. ambiens, dorsal (iliac) head	1793	0.044	0.3941	15
FMTL	M. femorotibialis lateralis	1434	0.088	0.1746	15
FMTIM	M. femorotibialis intermedius	1706	0.084	0.1863	25
FMTM	M. femorotibialis medialis	1089	0.089	0.0603	30
ILFBa	M. iliofibularis (cranial part)	1254	0.176	0.2134	0
ILFBp	M. iliofibularis (caudal part)	1254	0.176	0.2733	0
ITCa	M. iliotrochantericus caudalis (cranial part)	897	0.064	0.0469	25
ITCp	M. iliotrochantericus caudalis (caudal part)	897	0.064	0.038	25
IFE	M. iliofemoralis externus	479	0.025	0.0667	25
ITM	M. iliotrochantericus medius	181	0.058	0.0241	0
ITCR	M. iliotrochantericus cranialis	330	0.053	0.0488	10
IFI	M. iliofemoralis internus	410	0.041	0.0533	0
FCM	M. flexor cruris medialis	1109	0.036	0.435	35
FCLP	M. flexor cruris lateralis pars pelvica	544	0.24	0.2449	0
ISF	M. ischiofemoralis	419	0.033	0.0816	15
PIFML	M. puboischiofemorales medialis + lateralis	816	0.089	0.1669	15
OM	M. obturatorius medialis	3124	0.055	0.1651	25
CFP	M. caudofemoralis pars pelvica (et caudalis)	1125	0.108	0.215	15
GL	M. gastrocnemius pars lateralis	1836	0.12	0.5818	20
GIM	M. gastrocnemius pars intermedius	798	0.125	0.507	15
GM	M. gastrocnemius pars medialis	3124	0.094	0.5957	20
FL	M. fibularis longus	2270	0.081	0.9633	20
FDL	M. flexor digitorum longus	1130	0.048	1.0366	20
FPPD3	M. flexor perforans et perforatus digitorum 3	1154	0.025	1.0737	30
FPD3	M. flexor perforans digitorum 3	3210	0.017	1.02	35
FPD4	M. flexor perforans digitorum 4	1434	0.026	1.004	20
FHL	M. flexor hallucis longus	469	0.04	1.0939	25
EDL	M. extensor digitorum longus	833	0.049	0.8512	30
TCf	M. tibialis cranialis (femoral head)	686	0.045	0.4791	25
TCt	M. tibialis cranialis (tibial head)	686	0.045	0.4215	25

Six additional actuators were used to compensate for residual forces and moments at the pelvis during the motion and eight torque actuators—one for each DOF in the right limb—were used to compensate for mechanical work that could not be satisfied by the muscles alone (reserve actuators). Each optimization was tasked with minimizing the use of these reserve actuators, ensuring that, at each joint, the required joint moments were satisfied primarily through muscle force.

### 2.2. Simulations

Three simulations were generated from the experimental walking data. Each simulation used the same experimental data and musculoskeletal model as inputs, but used a different optimization framework to estimate MTU excitation, force and length changes. Three additional simulations were then generated from the experimental running data using the same model and optimization frameworks (table 1).

Simulations were first generated using the SO routine included in OpenSim. SO determines MTU excitation patterns by optimizing a predetermined objective criterion subject to the biomechanical constraints associated with the motion. The objective criterion used here minimized muscle activation squared, summed across all muscles at each time step [33]2.1
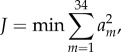
where *a_m_* is the activation level of the *m*th muscle. The time step was set to 0.005 s and MTU excitation, force and length time histories were obtained over the gait cycle. MTU force calculations included intrinsic muscle force–length–velocity relationships [45]. Because each time step is solved independently within the SO framework, there is neither energy transfer between time steps (e.g. tendon energy storage and return) nor muscle excitation–activation dynamics. Passive fibre force generation is also ignored, and tendons are assumed rigid with all MTU length changes occurring in the muscle fibres.

The second optimization framework used to generate simulations was OpenSim's CMC routine [42]. CMC is a hybrid forward–inverse approach, with muscle excitations for each time step determined using the same objective criterion as the SO routine. However, like purely forward dynamic simulations, the model state from a previous time step (e.g. joint angles, muscle activation level, tendon strain) influences the optimal solution for the current step. Because time steps are linked, this approach incorporates muscle excitation–activation dynamics and non-rigid tendon characteristics. Passive muscle fibre force generation is also accounted for.

In order to reduce the potentially confounding factors of different tendon and muscle models when directly comparing between SO and CMC, a third optimization framework was implemented. This approach was identical to the previous CMC framework, with the exception that, like SO, a rigid tendon model was implemented, and muscle passive force generation was removed. Using rigid tendons eliminates tendon–muscle fibre dynamics and partially negates the ability of a forward dynamics optimization to account for time-dependent muscle interactions (e.g. tendon energy storage and return). As a result, using this framework would not be a realistic choice under normal circumstances. However, eliminating these potentially confounding factors allows for a more direct comparison between the SO and CMC frameworks.

### 2.3. Experimental data

Experimental data for a representative walking (1.2 ms^−1^; 0.66 duty factor) and running trial (3.5 ms^−1^; 0.40 duty factor) were taken from a single adult bird (78.7 kg) of a previously collected dataset [2,8,39]. Three-dimensional segment and joint kinematics were calculated from retro-reflective marker clusters located on the pelvis, right-side femur, tibiotarsus and tarsometatarsus, and a single marker on digit III. Marker locations were recorded at 200 Hz using high-speed video (Peak Performance; Centennial, CO). Ground reaction forces were simultaneously collected using a Kistler force plate (model 9865E, Kistler, Winterthur, Switzerland). Data were filtered in OpenSim using a low-pass frequency of 10 Hz. Because only right-side data were collected experimentally, left-side motion and force data were estimated by mirroring the right-side data about the sagittal plane and phase-shifting the data 180° to generate a complete gait cycle.

### 2.4. Analysis

In each simulation, muscle excitation onset and offset timings were determined from the predicted muscle excitation patterns, with muscles considered to be excited when the values exceeded a 0.1 (i.e. 10% of maximum excitation) threshold. A period of excitation was then determined by first identifying the onset time as the closest previous time step where excitation fell below 0.05. Offset time was then identified as the first subsequent point that excitation fell below 0.05. Stance (i.e. foot in contact with the ground) and swing phases were identified and timing values were used to group muscles into ‘stance’ or ‘swing’ groups. Predicted muscle excitation onset and offset times were then normalized to the entire gait cycle and compared with existing avian EMG data [22,23] as a form of indirect validation.

MTU force and length time histories were used to generate comparisons among the six simulations. First, average muscle forces were calculated as the mean force value during stance and swing. An ‘integrated activation’ (iAct) value was also calculated for the two phases. To calculate iAct, the stance and swing phases were first normalized to per cent phase. The activation trajectory was then integrated over the entire phase to generate a single activity value ranging from 0 (no activity) to 100 (maximally active over the entire phase). Net MTU work was calculated for each muscle from the instantaneous MTU force and velocity values over the entire gait cycle. Positive and negative work were calculated for stance and swing by integrating only the positive and negative portions of the power curves of each MTU within each phase. Muscles were grouped based on anatomical location, creating seven distinct groups: (i) hip rotators (ITCa, ITCp, ITCR, ITM), (ii) biarticular hip–knee (ILa, ILp, ILFBa, ILFBp, FCLP, FCM), (iii) knee extensors (FMTL, FMTIM, FMTM), (iv) gastrocnemius (GL, GIM, GM), (v) digital flexors (FDL, FHL, FL, FPPD3, FPD3, FPD4), (vi) ankle flexors (EDL, TCf, TCt) and (vii) other (proximal) muscles (OM, IFE, IFI, ISF, PIFML, CFP, AMB1, AMB2, IC).

To evaluate the influence that reserve actuators may have had on simulation results, average and peak reserve actuator values were compared with the peak net joint torques (obtained via OpenSim's inverse dynamics analysis). Reserve actuator work was also calculated from the actuator torque and joint angle trajectories, analysed in the same manner as MTU work and then compared with the total amount of mechanical work generated by the muscles in each corresponding simulation. In addition, for the CMC simulations, which were not explicitly constrained to follow the experimental joint kinematics, root mean square (RMS) differences between the experimental and simulation joint kinematics were calculated for the entire movement.

## 3. Results

The three optimization frameworks were able to successfully generate simulations of walking and running, with all six simulations generating a solution. In the CMC simulations, peak errors in simulated joint trajectories were within 2° of experimental angles and RMS errors well below 0.1° (see electronic supplementary material, table S1).

### 3.1. Reserve actuators

In all six simulations, average reserve actuator values remained below 10% of the inverse dynamics moment with the exception of hip ad–abduction, knee ad–abduction and ankle flexion–extension (table 3, average reserve torque). Knee ad–abduction was below 10% for all simulations but *W*_CMCC_ (15%). Hip ad–abduction had by far the highest average reserve actuator values, accounting for up to 90% of the inverse dynamics moment. Average ankle flexion–extension moments were consistent between all simulations, ranging from 9.1 to 16.3%. Peak reserve actuator values were more variable across the different simulations. Peak knee rotation and knee flexion–extension reserve values fell below 10% of the inverse dynamics torques in all simulations except for *W*_CMCC_. Peak hip flexion–extension reserve values were below 10% in all but *R*_SO_ (12%) and *R*_CMCR_ (15%). Peak hip rotation reserve actuator values all fell below 15%. Ankle flexion–extension and MTP flexion–extension peak reserve values were high in most of the simulations. The hip ad–abduction reserve actuator was highest in all six simulations (table 3).

**Table 3.** Average and peak moments as well as net mechanical work generated by the reserve actuators for each of the six simulations. Shaded columns are for the three walking simulations. Moment values are presented in Nm and parenthetical values indicate the per cent of the inverse dynamic analysis joint torque. Work values are presented in joules (J) and parenthetical values are percentages relative to the total muscle–tendon unit mechanical work generated in each simulation. Positive values indicate hip/knee extension, adduction and medial rotation, and ankle/MTP flexion moments. Positive/negative mechanical work indicates energy being added/removed from the limb.

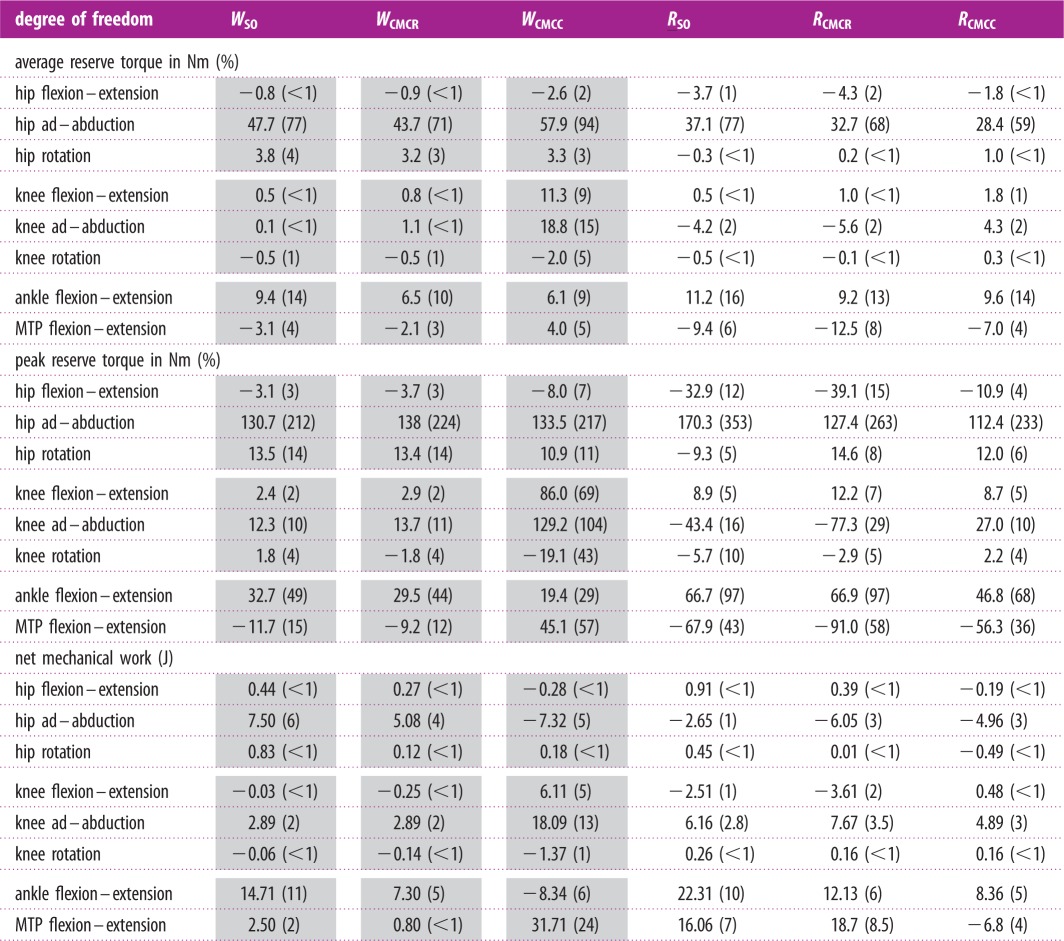

**Figure 2.**
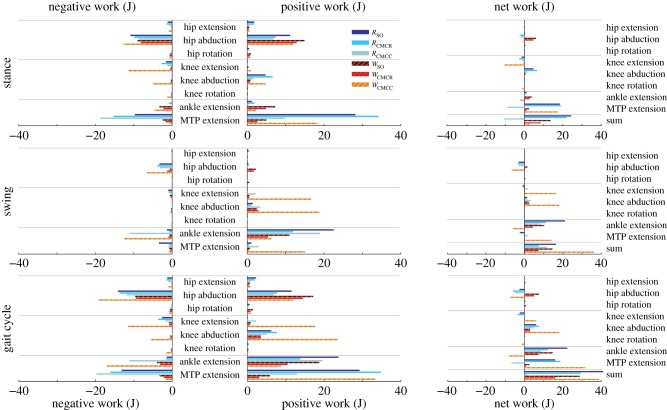
Positive, negative and net mechanical work generated by the reserve actuators in each simulation. Positive/negative work indicates energy (joules) added to/removed from the limb during the movement. Sum: total of all reserve actuators.

Even though the hip ad–abduction reserve actuator had the highest average and peak reserve actuator values, its contribution to limb mechanical work over the gait cycle was small (less than 6% of total muscle work) in all simulations (table 3 and figure 2). Knee ad–abduction reserve actuator work was consistently positive, with values ranging from 2.89 (2%; *W*_CMCR_, *W*_SO_) to 18.09 J (13%; *W*_CMCC_). The highest net values were generated by the ankle and MTP reserve actuators, with magnitudes reaching 31.71 J (24%; table 3 and figure 2). The other reserve actuators had low net mechanical work (less than 5%) over the simulation.

### 3.2. Muscle excitation and activation

**Figure 3.**
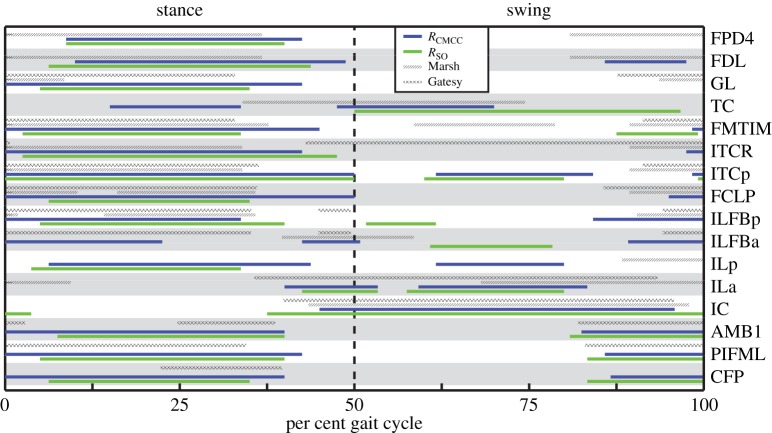
Example simulated muscle excitation timings during running. Blue (dark grey) and green (light grey) bars indicate periods of excitation for the RSO and RCMCC solutions, respectively. For comparison, onset and offset timing obtained from EMG studies of guinea fowl during slower [20] (Gatesy, 1.0 m s^−1^, hatched bars) and faster [21] (Marsh, 1.5 m s^−1^, striped bars) running are provided. Owing to differences in stance and swing times between the studies, stance and swing phases were normalized to 50% of the gait cycle. Zero per cent (0%) of gait cycle indicates the beginning of the stance phase. The other four simulations had similar excitation patterns.

Muscle timing data were similar across all six simulations, with the majority of muscles having a single excitation period that occurred primarily in either stance or swing (figure 3). The major hip, knee and ankle extensors (e.g. M. flexor cruris lateralis pars pelvica, FCLP; M. femorotibialis, FMTIM; M. gastrocnemius, GL), many hip rotators (e.g. Mm. iliotrochantericus, ITCp, ITCr) and the digital flexors (M. flexor digitorum longus, FDL) were primarily excited during stance. The uniarticular hip extensors, M. caudofemoralis pars pelvica (CFP) and M. puboischiofemoralis (PIFML) were excited from mid-to-late swing through mid-stance. Owing to their large origin sites, the M. iliotibialis lateralis and M. iliofibularis were partitioned into cranial and caudal regions in the model. In both muscles, the caudal portions (ILp, ILFBp) tended to be excited during stance whereas the cranial portions (Ila, ILFBa) were excited during swing (figure 3). The hip and ankle flexors (e.g. M. iliotibialis cranialis, IC; M. tibiocranialis, TC) were primarily excited during swing. In both running and walking ISF is not excited. IFE, IFI and FHL are only excited during the running simulations.

Although no ostrich EMG data are available for direct comparison, simulation results compare favourably to previous comprehensive studies of guinea fowl limb muscle activity (figure 3; [22,23]). Except for small timing changes that are to be expected owing to comparisons being performed between different avian species, the simulated muscle excitation patterns were consistent with the empirical data: most muscles had a single period of EMG activity in either the stance or swing phase. Nonetheless, there were a few notable exceptions. Similar to EMG recordings [23], CFP was excited during mid-stance. However, either an additional period of excitation or an extended single period occurred during late swing in the simulations that was not evident in the EMG data. The CFP may have been preferentially used to slow down hip flexion and assist in hip extension prior to foot strike. Digital flexor and ankle extensor onset times occurred in early stance in the simulations, but EMG recordings suggest an earlier onset during late swing (e.g. FPD4, FDL, GL). Last, EMG recordings for the ITCR suggest that this muscle is excited during swing. However, the simulations consistently excited ITCR during mid-stance, likely to oppose the high hip lateral rotation moment. Instead, ITCp was excited during both mid-swing and stance in the simulations, whereas EMG data indicate that this muscle only has a single excitation period beginning in late-swing through stance. The ITCa, ITCR and ITCp are all medial hip rotators and discrepancies could be owing to comparing different species. This will remain uncertain until ostrich EMG data become available, even though EMG patterns in avians measured to date generally are conservative [23,50].

**Figure 4.**
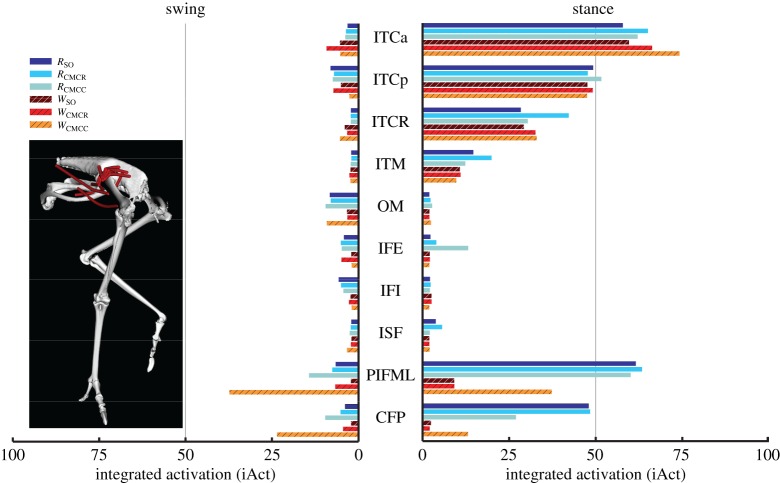
Integrated muscle activation values of the uniarticular hip muscles during the swing and stance phases for each of the six simulations. Solid bars, running simulations; striped bars, walking simulations.

**Figure 5.**
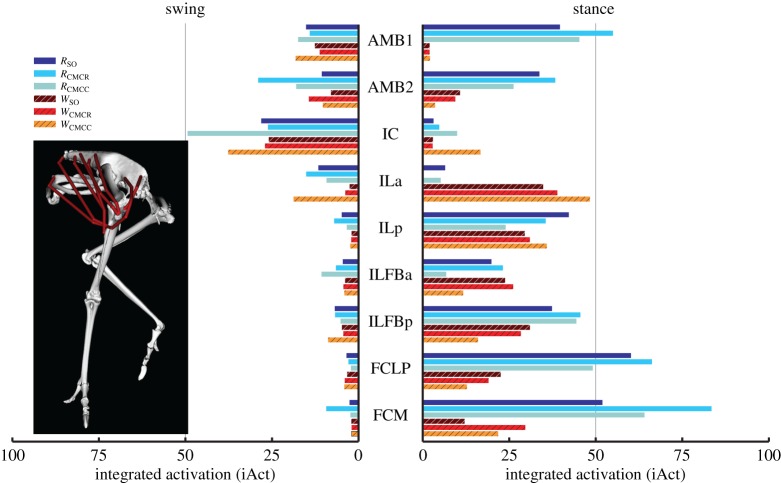
Integrated muscle activation values of the biarticular muscles crossing the hip and knee during the swing and stance phases for each of the six simulations. Solid bars, running simulations; striped bars, walking simulations.

When averaged across all muscles, iAct was always greater during stance than swing in both gaits, with the smallest difference occurring in *W*_CMCC_ (21.2 versus 16.7). The running simulations also consistently required more muscle activity than during walking (e.g. *R*_CMCC_, 21.5; *W*_CMCC_, 19.6). In both gaits, the PIFML and CFP muscles were active during both phases. However, stance phase iAct was much larger during running than walking (figure 4). The medial hip rotators ITCa, ITCp, ITCR and ITM and the lateral hip rotator OM had similar activity levels in all simulations, with the medial rotators primarily active during stance and OM active during swing. Conversely, many of the biarticular muscles crossing the hip and knee (i.e. ILp, ILFBp, FCLP, FCM) had noteworthy changes in iAct between the two gaits (figure 5). Even though muscle activity primarily occurred during stance for both gaits, iAct values for ILFBp, FCLP and FCM were markedly lower in the walking motion. Similar to their excitation patterns, ILa and ILFBa had notable iAct values during both the stance and swing phases in running (figure 5). AMB1 and AMB2 had similar activity levels during swing in both gaits, but had increased activity during stance in running. The IC, a hip flexor and knee extensor, had consistent iAct values across all simulations, which were highest during swing.

**Figure 6.**
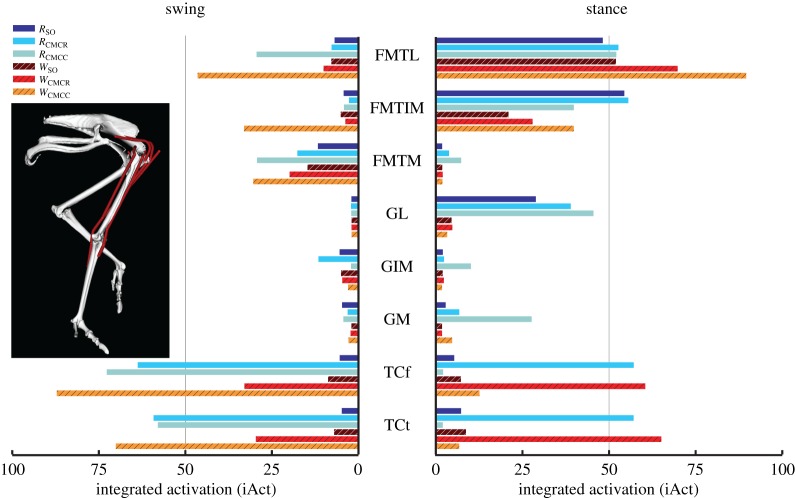
Integrated muscle activation values for the uniarticular and biarticular knee and ankle muscles during the swing and stance phases for each of the six simulations. Solid bars, running simulations; striped bars, walking simulations.

**Figure 7.**
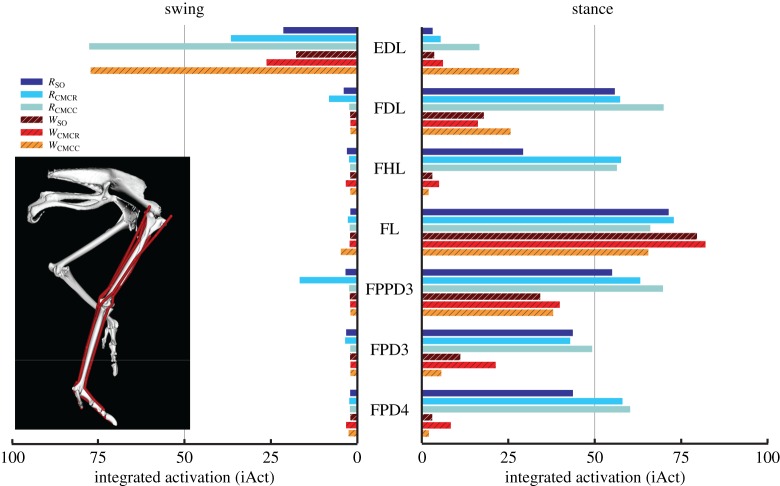
Integrated muscle activation values for the muscles crossing the metatarsophalangeal (MTP) joint during the swing and stance phases for each of the six simulations. All of these muscles are either biarticular (ankle–MTP) or multiarticular (knee–ankle–MTP). Solid bars, running simulations; striped bars, walking simulations.

In both gaits, the uniarticular knee extensors FMTL and FMTIM had larger iAct values during stance than swing, whereas the converse was true for FMTM (figure 6). Knee extensor iAct values differed greatly between simulations, with the CMC compliant tendon simulations (i.e. *W*_CMCC_ and *R*_CMCC_) producing higher values during swing compared with the other four simulations. The major ankle extensors (Mm. gastrocnemius: GL, GIM, GM) had higher integrated muscle activity during stance in the running simulations. Ankle flexor (TCf, TCt) iAct was comparable between running and walking (e.g. figure 6, TCf: *W*_SO_ versus *R*_SO_). However, the CMC simulations consistently estimated higher overall ankle flexor activity than the SO simulations, with the greatest differences occurring during swing in the CMC rigid tendon simulations. Digital flexor (FPPD3, FPD3, FPD4, FDL) muscle activity occurred almost exclusively during stance (figure 7). Differences in FPPD3, FPD3 and FPD integrated activity occurred between the two gaits, with running simulations consistently having higher values. The digital extensor EDL was primarily active during swing but did have a small amount of activity during stance.

### 3.3. Muscle force and work

**Figure 8.**
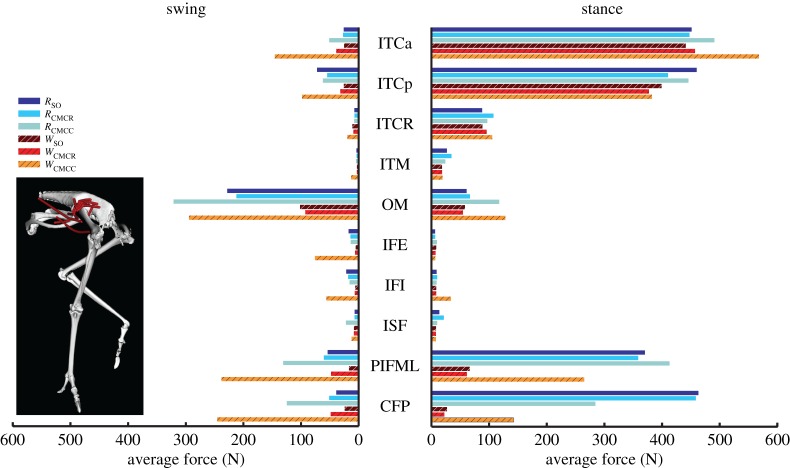
Average muscle force values of the uniarticular hip muscles during the swing and stance phases for each of the six simulations. Solid bars, running simulations; striped bars, walking simulations.

**Figure 9.**
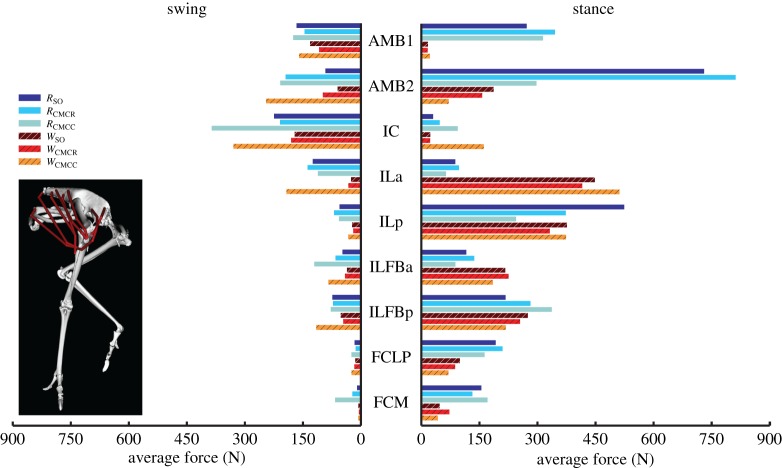
Average muscle force values of the biarticular muscles crossing the hip and knee during the swing and stance phases for each of the six simulations. Solid bars, running simulations; striped bars, walking simulations.

**Figure 10.**
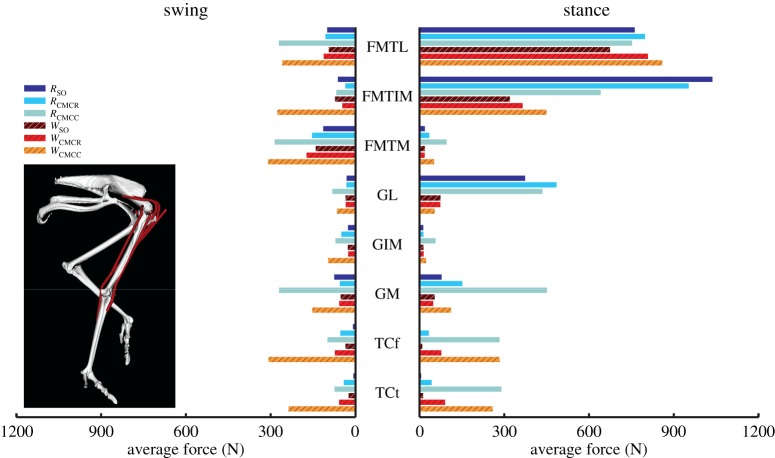
Average muscle force values for the uniarticular and biarticular knee and ankle muscles during the swing and stance phases for each of the six simulations. Solid bars, running simulations; striped bars, walking simulations.

**Figure 11.**
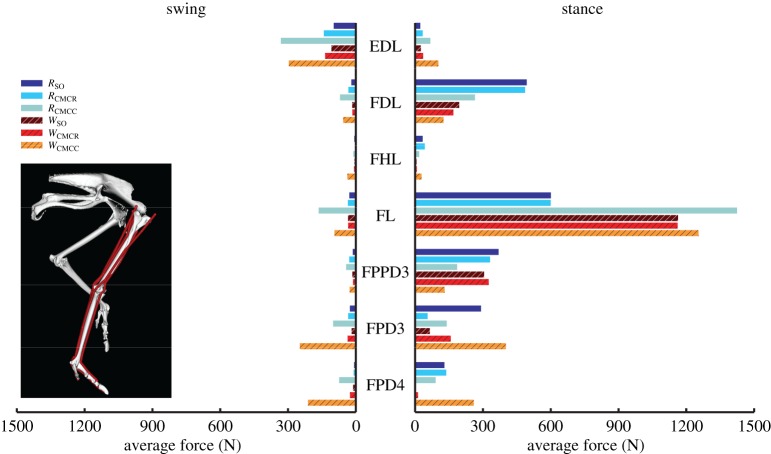
Average muscle force values for the muscles crossing the metatarsophalangeal (MTP) joint during the swing and stance phases for each of the six simulations. All of these muscles are either biarticular (ankle–MTP) or multiarticular (knee–ankle–MTP). Solid bars, running simulations; striped bars, walking simulations.

Average muscle forces tended to follow the same trends as activation, but there was higher variability between optimization frameworks, with the compliant tendon simulations using CMC (*R*_CMCC_, *W*_CMCC_) regularly generating larger forces than the other simulations (figures 8–11). Among all the uniarticular hip muscles, the medial hip rotators and the hip extensors (PIFML, CFP) had the greatest forces during stance (figure 8). During swing, the PIFML and CFP had large forces in the compliant tendon CMC simulations. The lateral hip rotator OM consistently had larger forces in running. Except for the AMB1 and AMB2 muscles—which clearly generated more force during running—the biarticular hip–knee muscles had similar amounts of force in both gaits (figure 9). Swing phase forces were consistent across simulations and movements, with the IC, AMB1 and AMB2 muscles generating the largest average forces. The uniarticular knee extensors FMTL and FMTIM and the digital flexor FL had the greatest forces during stance (figures 10 and 11). The GM and GL had large average stance forces in running, but much lower values in walking. The ankle flexors (TCf, TCt) had small forces during both stance and swing in the CMC simulations, with the compliant tendon simulations generating the highest average forces (figure 10). Digital flexor muscles' forces had a clear distinction between stance and swing, with much smaller swing forces compared with stance (figure 10). The digital extensor EDL primarily generated force during swing.

**Figure 12.**
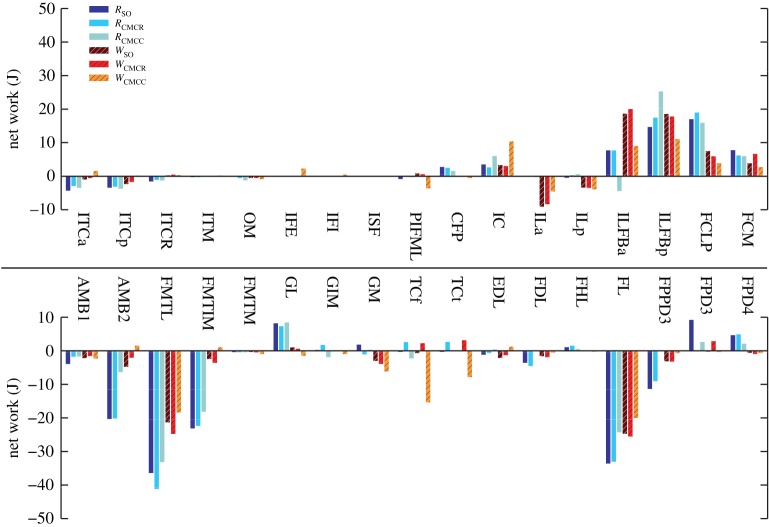
Net mechanical work for each musculotendon unit over an entire gait cycle. Positive/negative work indicates work added/removed from the biomechanical system. Solid bars, running simulations; striped bars, walking simulations.

Total MTU mechanical work had similar patterns between walking and running (figure 12). The hip rotators (ITCa and ITCp), knee extensors (FMTL and FMTIM), AMB2, FL and FPPD3 consistently produced negative work, whereas many of the biarticular hip extensors (e.g. ILFB, FCLP, FCM), the hip flexor IC, and ankle extensor (GL) generated positive work in the simulations. In contrast, the mechanical work generated by the ankle flexors TCf and TCt varied greatly between simulations, with no clear pattern. The remaining muscles tended to generate little positive or negative net mechanical work (figure 12).

**Figure 13.**
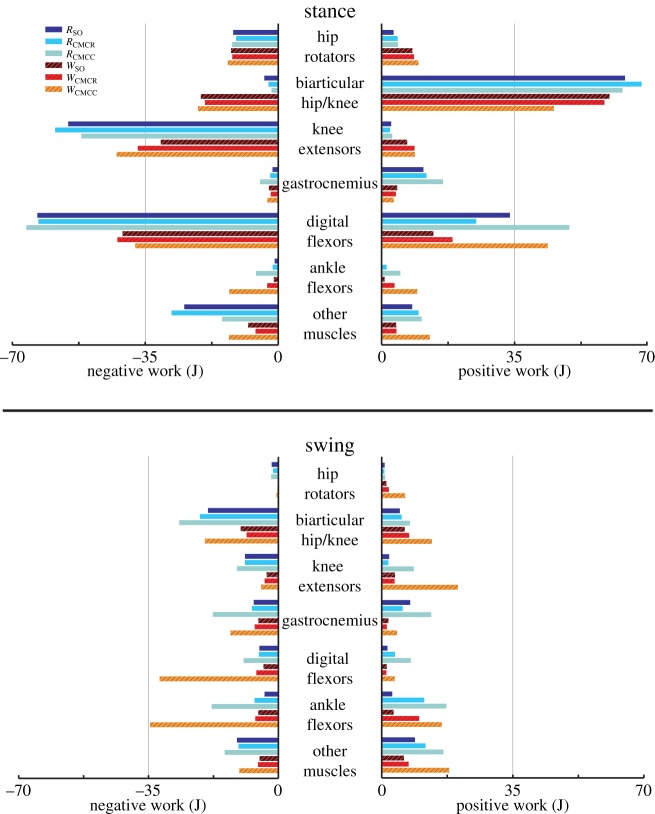
Positive and negative musculotendon work generated by different muscle groups over the stance and swing phases of a gait cycle. Positive/negative work indicates work added/removed from limb and were calculated from the corresponding portion of the power curves. Muscles were grouped as either hip rotators (ITCa, ITCp, ITCR, ITM), biarticular hip/knee (ILa, ILp, ILFBa, ILFBp, FCLP, FCM), knee extensors (FMTL, FMTIM, FMTM), gastrocnemius (GL, GIM, GM), digital flexors (FDL, FHL, FL, FPPD3, FPD3, FPD4), ankle flexors (EDL, TCf, TCt) or other muscles (OM, IFE, IFI, ISF, PIFML, CFP, AMB1, AMB2, IC). Solid bars, running simulations; striped bars, walking simulations.

The total amount of positive and negative muscle work generated during swing was much lower than that generated during stance (figure 13). There were increases in both positive and negative mechanical work generated by the M. gastrocnemius, digital flexors and ankle flexors in *W*_CMCC_ and *R*_CMCC_ relative to the other simulations. During stance, the biarticular hip–knee muscles generated the majority of the positive work in both gaits, amounting to more than twice their negative work (figure 13). The digital flexors generated large amounts of both positive and negative work, with similar amounts of negative work predicted by all six simulations. However, the amount of positive work generated by the digital flexors increased dramatically in *R*_CMCC_ and *W*_CMCC_ simulations. On the other hand, the knee extensors generated a large amount of negative work and very little positive work. The gastrocnemius group generated very little work in walking, but consistently produced a small amount of positive work in running.

### 3.4. Muscle functional roles

To act as a motor that drives motion, muscles must produce force during concentric contractions and generate positive work. In both gaits, the muscles identified as motors were the same (table 4). The IC and AMB2 provided much of the energy required during swing, whereas the biarticular hip and knee muscles (ILFBa, ILFBp, FCM, FCLP) and lateral gastrocnemius (GL) provided energy during stance (figures 11 and 12 and table 3). In contrast, the hip rotators (ITCa, ITCp, ITM, ITCR), FMTM, AMB1, ankle flexors (TCf, TCt, EDL) and uniarticular hip extensors (PIFML, CFP) all acted as struts, generating moderate to high forces but little positive or negative work. Furthermore, the digital flexors acted primarily as springs during stance, first absorbing energy (negative work) in early stance and then generating positive work during late stance (figure 13 and table 4). Finally, the FDL also generated force during an eccentric contraction in early stance, resulting in net negative limb work (i.e. a brake). Likewise, the knee extensors FMTM and FMTL acted as brakes, absorbing energy from the limb during stance (figure 13 and table 4). A few differences in functional roles between gaits were evident. During walking, the IL and GM acted as brakes and absorbed energy from the limb during stance. However, these muscles acted primarily as struts during running, generating force but very little work. Muscles with a second excitation period during running did not alter the functional roles of the comparable excitation periods between the two gaits. Instead, the additional excitation periods added an additional role to the muscle during the movement. The AMB1 and AMB2 had additional roles as a strut and brake, respectively, during stance in running, whereas the ITCa and ILFBa had additional roles of strut and brake, respectively, during swing.

**Table 4.** Muscle functional roles based on muscle–tendon unit excitation, force and mechanical work. Differences in roles between walking and running are shown in italics. Muscles performing roles in both swing and stance have roles that are separated by a colon (:) with their role in swing first (e.g. AMB2 acts as a motor during swing, then acts as a brake during stance).

muscle	abbreviation	classification		primary role	
		running	walking	running	walking
M. iliotibialis cranialis	IC	swing	swing	motor	motor
M. iliotibialis lateralis (cranial part)	ILa	*swing*	*stance*	*strut*	*brake*
M. iliotibialis lateralis (caudal part)	ILp	stance	stance	*strut*	*brake*
M. ambiens, ventral (pubic) head	AMB1	*both*	*swing*	strut : strut	strut
M. ambiens, dorsal (iliac) head	AMB2	*both*	*swing*	motor : brake	motor
M. femorotibialis lateralis	FMTL	stance	stance	brake	brake
M. femorotibialis intermedius	FMTIM	stance	stance	brake	brake
M. femorotibialis medialis	FMTM	swing	swing	strut	strut
M. iliofibularis (cranial part)	ILFBa	both	stance	brake : motor	motor
M. iliofibularis (caudal part)	ILFBp	stance	stance	motor	motor
M. iliotrochantericus caudalis (cranial part)	ITCa	stance	stance	strut	strut
M. iliotrochantericus caudalis (caudal part)	ITCp	*both*	*stance*	strut : strut	strut
M. iliofemoralis externus	IFE	*stance*	*off*	strut	
M. iliotrochantericus medius	ITM	stance	stance	strut	strut
M. iliotrochantericus cranialis	ITCR	stance	stance	strut	strut
M. iliofemoralis internus	IFI	*swing*	*off*	strut	
M. flexor cruris medialis	FCM	stance	stance	motor	motor
M. flexor cruris lateralis pars pelvica	FCLP	stance	stance	motor	motor
M. ischiofemoralis	ISF	off	off		
M. puboischiofemorales medialis + lateralis	PIFML	stance	stance	strut	strut
M. obturatorius medialis	OM	swing	swing	strut	strut
M. caudofemoralis pars pelvica (et caudalis)	CFP	stance	stance	strut	strut
M. gastrocnemius pars lateralis	GL	stance	stance	motor	motor
M. gastrocnemius pars intermedius	GIM	stance	stance	strut	strut
M. gastrocnemius pars medialis	GM	stance	stance	*strut*	*brake*
M. fibularis longus	FL	stance	stance	brake	brake
M. flexor digitorum longus	FDL	stance	stance	spring	spring
M. flexor perforans et perforatus digitorum 3	FPPD3	stance	stance	spring	spring
M. flexor perforans digitorum 3	FPD3	stance	stance	spring	spring
M. flexor perforans digitorum 4	FPD4	stance	stance	spring	spring
M. flexor hallucis longus	FHL	*stance*	*off*	spring	
M. extensor digitorum longus	EDL	swing	swing	strut	strut
M. tibialis cranialis (femoral head)	TCf	swing	swing	strut	strut
M. tibialis cranialis (tibial head)	TCt	swing	swing	strut	strut

## 4. Discussion

Combining detailed musculoskeletal models and simulations with empirical data allows for the estimation of quantities that can greatly enhance our understanding of specific functional roles during dynamic movements [28,29,51]. Although anatomical and EMG studies can provide insight into muscle classification relative to gait events (e.g. stance versus swing phase), a detailed understanding of a muscle's functional role(s) requires additional quantities that are not readily obtained using experimental techniques. The musculotendon force and mechanical work data generated in this study enable the determination of specific muscle mechanical roles such as motor, brake, strut or spring during gait [19–21]. These roles provide important information regarding how energy flows through the limb and generates the required external work during movement. Muscle functional roles were also mainly insensitive to optimization approach or gait type (table 4).

However, there were some subtle differences between the SO and computed muscle control compliant tendon (CMCC) simulations, especially among muscles with long tendons that were classified as mechanical springs (table 4). These differences were most evident in the digital flexors (FL, FPPD3, FPD3, FPD4) during running, where the magnitude of the net mechanical work produced by these muscles was lower in CMCC than SO (figure 12). On the other hand, the amount of negative and positive work generated by these muscles in CMCC was higher than SO (figure 13). Ideal mechanical springs have zero net mechanical work; all absorbed energy is stored and returned. An MTU acting in a spring-like fashion will exhibit high amounts of positive and negative work but have a low net mechanical work. Although the digital flexors exhibited these spring-like characteristics in both optimization approaches, the CMCC simulations more clearly indicated that the muscles were acting as springs. Using CMCC may be more helpful in other situations, where functional roles are not as easily identified.

For example, the ankle flexor group produced close to zero net mechanical work during stance in all simulations (figure 13). The total negative and positive mechanical work varied greatly between simulations, however. Positive and negative mechanical work were near zero in the SO simulations, defining these muscles as struts during stance. However, the positive and negative values were many times greater in the CMCC simulations, resulting in a functional role of a spring for these muscles (figure 13). Based on their anatomical features (i.e. short muscle fibres and long tendons), it is likely that the ankle extensor MTUs truly do act as springs as suggested by the CMCC simulations. Interestingly, the computed muscle control simulations incorporating a rigid tendon (CMCR) generated results similar to the SO simulations. Thus, the incorporation of both (i) a flexible tendon and (ii) the ability to account for energy storage and return may be important when inferring whether a muscle acts as a strut or spring.

Relative to the hip, the knee undergoes greater joint excursions during walking and running in birds. As a result, studies of avian gait have historically characterized muscles crossing the knee as critical to driving movement [52]. On the other hand, models of human walking and running have found muscles crossing the knee to primarily act as brakes, absorbing energy during stance [53,54]. Ostriches are uniquely situated—as birds they likely use similar mechanics to smaller cursorial birds but are larger in size and thus may require similar mechanics to larger bipedal animals such as humans. An examination of muscular roles provides evidence that ostrich gait is at least partly hip-driven, with the major biarticular hip-to-knee muscles acting as motors and generating much of the positive work in both gaits (table 4 and figure 12: ILFB, FCLP, FCM). Bi-articular muscles are thought to act primarily to transfer energy across joints (i.e. as a strut) and the function of the ostrich bi-articular hip extensors as a motor may be greater than previously inferred. In contrast, despite generating large forces, the uniarticular hip extensors (PIFML, CFP) had mechanical work values near zero and acted as struts. This result is consistent with previous inverse dynamics analyses (i.e. joint-level analyses) that predict little hip joint work [2]. However the muscle-level analysis performed here, which includes work done by multi-joint muscles, shows that total hip muscle work may be disproportionate to joint-level estimates and suggests that ostriches may use more complex hip–knee interactions than humans to drive their limbs. The major knee extensors (FMTL, FMTIM) acted as brakes during stance, suggesting that ostriches, like humans, employ these muscles to assist in maintaining whole-body stability. Of the muscles active in swing, only IC and AMB2 acted as motors, indicating that these muscles are the key drivers of swing phase mechanics, especially limb protraction.

Avian distal limb muscles are remarkably specialized, consisting of extremely long tendons that have high energy storage and return potential [2,15,17,18]. In this study, regardless of the type of simulation, the lateral gastrocnemius (GL) and digital flexors generated large but nearly equal amounts of negative and positive work, resulting in near zero net mechanical work in both gaits (figures 7 and 11–13). These muscles acted as springs, first absorbing energy during early stance and then returning this energy during late stance. The magnitudes of positive and negative work generated by these MTUs were also greater during running than walking (e.g. −66.5 versus −37.8 J and 49.6 versus 44.0 J; *R*_CMCC_ versus *W*_CMCC_), congruent with the notion that these MTUs are acting as springs that use tendon energy storage and return (figure 13). The increased distal limb muscle activity and work observed in the running simulations is consistent with the widely held notion that ostriches increase their reliance on these specialized elastic structures during higher speed movements to improve running economy [2,17].

In both gaits, individual muscle excitation timing and integrated muscle activity occurred primarily during either stance or swing, suggesting that primary muscle functional roles may be associated with gait phases (figures 3–7). These data allowed for general muscle classification, which was found to be insensitive to simulation type and generally consistent between the two gaits; only seven of the 34 muscle actuators had gait-specific classifications (table 4). In all seven muscles with gait-specific classifications, the running gaits had additional excitation periods that were not observed in the walking simulations. For example, AMB1 was excited during swing in both gaits. In running, AMB1 also had an additional excitation period during stance (figure 5). These findings may be due to the higher mechanical demands associated with running and muscles may take on additional roles to assist with meeting these demands.

Although a broad division based on gait phases could be identified for individual muscles, this division did not scale to anatomical groups. For example, within the femorotibialis muscle group, FMTM and FMTL were classified as stance phase muscles but FMTIM was classified as a swing phase muscle based on excitation timing. Similarly, the cranial portions of M. iliofibularis (ILFBa) and M. iliotibialis lateralis (ILa) had different classifications from the caudal portions (ILFBp, ILp) during running (table 4). Previous EMG studies have also suggested that muscles within anatomical groups are differentially excited. Marsh *et al*. [22] showed that the Mm. femorotibialis and M. iliofibularis usually had two excitation periods during running—one during stance and a second during swing. Gatesy [23] also found the cranial and caudal compartments of the M. iliotibialis lateralis to have distinct activity patterns. Our study, combined with the previous EMG work, highlights the need to exercise caution when assuming that anatomically similar muscles also have similar functions during movement. In addition, the present results further suggest that even general classification of muscles based solely on excitation relative to stance or swing phase mechanics may be too simplistic. For example, despite their primary activity being clearly associated with either stance or swing, many limb muscles in this study also had small amounts of excitation over transition regions (e.g. late stance to early swing) [22,43]. The reasons for this low level excitation are less clear: activity may be associated with a secondary minor functional role or may be a result of time delays between muscle activity and force generation—future work directed at resolving this uncertainty (e.g. combining simulations with induced acceleration and/or segment power analyses [55–57]) is warranted.

When constructing optimizations designed to reproduce experimental data, OpenSim allows the user to apply ‘reserve actuators' to each joint in the model to compensate for any mechanical forces that could not be satisfied by the muscles alone. Because the optimization framework only uses these actuators when muscle forces are insufficient, the actuator values can provide a rough estimate of how experimental data and musculoskeletal model inaccuracies influence a simulation. During human movements, a threshold value of 5% of the net joint moments for reserve actuator values (average and peak) has been suggested as one indicator of a high-quality simulation [58]. In this study, average reserve actuators fell below 10% of net joint moments in 37 of the 48 cases (table 3, average). The most notable exception occurred in the average hip ad–abduction moment, which exceeded 50% in all six simulations. Peak values were more variable but hip ad–abduction, ankle and MTP flexion–extension reserve actuators were high in most of the simulations (table 3). One plausible reason for the high average and peak reserve actuator values is that they are compensating for unmodelled passive tissues and structures. Functionally, passive tissues act primarily as struts or springs, generating high forces but little mechanical work. To further assess whether the reserve actuators represent unmodelled passive structures, the positive, negative and net mechanical work generated by each actuator was calculated. Except for ankle and MTP flexion–extension, net mechanical work was generally low (i.e. less than 5% of the 134.7–224 J in total muscle work; table 3 and figures 2 and 12), suggesting that most reserve actuators likely represented passive structures.

Ostriches, like most birds, have remarkably few hip adductor muscles [9,59]. This is not surprising, because inverse dynamics analyses have shown that the intersegmental hip abduction moment is less than half the hip extension moment during stance in running [2]. However, many of the biarticular hip extensors and knee flexors, which are the main drivers during gait (table 4 and figure 13), also have large hip abduction moment arms. Therefore, these muscles generate a very large hip abduction moment during stance that cannot be counteracted by adductor muscles alone. Instead, passive mechanisms, such as bony contact between the femur and antitrochanter and strong ligaments [12–14] likely oppose this abduction moment. In our study, the hip ad–abduction reserve actuator was used to represent these passive mechanisms that are not explicitly modelled. Both the net mechanical work generated and the pattern of work generation exhibited by this reserve actuator were consistent with it representing passive tissues. During stance, this actuator generates an equal amount of negative and positive work, resulting in little net mechanical work during the modelled motions. In addition, the negative work associated with the hip ab–adduction actuator was generated during early stance and the positive work was generated during mid-to-late stance, consistent with the expected energetics of a passive structure that can stretch to absorb and then return energy (table 3 and figure 2).

To further test if the hip ad–abduction reserve actuator represented unmodelled passive tissues and better understand how these tissues may influence muscle coordination, a series of post hoc simulations using CMCC were generated in which the hip adduction reserve actuator was systematically reduced (i.e. reducing passive tissue contributions). As passive force contributions decreased, the muscle IC, despite acting as a hip flexor, was increasingly recruited during stance owing to its small hip adduction moment. After IC was maximally recruited, hip extension muscle activity was decreased to reduce the induced hip abduction moment by these muscles, replaced by increasing the torque generated by the hip extension reserve actuator. Both the recruitment of IC during stance, which has been found to be active exclusively during swing in other birds [22,23], and the increased reliance on the hip extension reserve actuator to power the motion suggest that passive hip structures are important during ostrich gait. The avian hip is an excellent example of a joint where non-muscular soft tissues and bony stops deserve careful consideration in dynamic analyses of locomotion.

However, rigorously implementing sufficiently accurate passive structures introduces additional challenges when building models and simulations. Rigid body contact models exist that could be implemented to model bony stops [60–63]. However, implementing these contact models is difficult as detailed information of both the underlying contact geometry and detailed joint motion data are necessary (i.e. subject-specific models), which are rarely available. In addition, contact models can be computationally expensive, especially when implemented at multiple joints, further increasing the time required to generate an optimal simulation. Similar constraints and limitations are associated with modelling other non-muscular passive tissues, where detailed knowledge of joint and tissue geometry is necessary. One alternative approach that has been used successfully in numerous human studies is to quantify the total passive behaviour of a joint using regression equations [64–66]. These equations are usually generated in the form of a net passive torque as a function of a single joint angle. However, creating these characteristic regression functions requires extensive cadaver-based work, especially when trying to characterize how the tissues interact between multiple DOF at a joint.

On the other hand, the ankle and MTP reserve actuators generated a substantial amount of positive work, suggesting that they did not represent passive structures but were compensating for muscle deficiencies. Peak MTP reserve actuator values occurred during mid-stance to assist the digital flexors, whereas peak ankle reserve values occurred during mid-swing to assist the ankle flexors. To confirm that muscle weakness was responsible for the simulations requiring these reserve actuators, an additional *R*_CMCC_ running simulation was performed in which the maximum isometric force of the digital flexors and ankle flexors was doubled. Doubling the strength of the digital flexors eliminated the need for the MTP reserve actuator, confirming that these muscles appear to be weak relative to the motion requirements. This result is consistent with findings in previous human running studies, where models of the plantar flexor muscles were incapable of generating sufficient torque to overcome the mechanical demands at the ankle joint [5,67]. Surprisingly, doubling the maximum isometric force of the ankle muscles did not reduce the required ankle flexion reserve torque—in fact, the required reserve torque was higher in this simulation. Further inspection revealed that the antagonistic digital flexors were passively generating force during mid-swing owing to muscle fibres operating at fibre lengths greater than the optimal fibre length. In the model, the ankle flexors cannot counteract these passive muscle forces using the current force ratio between the two muscle groups. In general, muscle fibre excursions tended to be larger than might be expected empirically, especially over regions where the joints also underwent large angle changes such as those found in swing (electronic supplementary material, figure S1). Lumped-parameter muscle models, like the Hill-type muscle model used here, tend to overestimate fibre excursions, which may explain why the digital flexors produce passive force during swing [45,68].

Despite these model inconsistences, all six simulations predicted overall muscle coordination patterns consistent with previously collected guinea fowl EMG data (figure 3, [20,21]). In addition, the percentage of muscle activity that occurs during swing (13.9–38.6%; see electronic supplementary material, table S2) compares favourably with previous muscle blood flow data suggesting that one quarter of the energetic cost of running occurs during swing in guinea fowl [22]. Combined with the good excitation timing comparisons in the vast majority of the muscles, these data indicate that the excitation patterns predicted by the simulations in this study are, in general, biologically reasonable and realistic. The high level of similarity between the predicted ostrich muscle coordination patterns and those of smaller cursorial birds also suggests that, despite experiencing a large change in size, ostriches appear to have conserved a gait coordination pattern inherited from a common avian ancestor, which is unsurprising given the apparent conservatism in avian pelvic limb muscle activity [23,50].

Although muscle functional roles were found to be insensitive to the three different optimization frameworks, there were some subtle differences in muscle quantities. During both walking and running, total muscle activity was consistently lower in the SO simulations than in both CMC simulations. This is most likely a direct result of the CMC simulations including excitation–activation dynamics, which can increase muscle co-contraction. The CMCC simulations also generated greater muscle forces despite having similar iAct values to the other simulations (e.g. figures 5 and 9; TCf, TCt), with differences likely due to the incorporation of fibre–tendon dynamics that create substantial changes in the force generation properties of muscle. Caution should be taken when eliminating muscle–tendon dynamics from biomechanical analyses, especially when investigating specific muscle quantities, motions that require large changes in joint motion, or muscles with relatively long tendons. Further tests against a gold standard (i.e. muscle fibre length measurements obtained via sonomicrometry or tendon force measurements via tendon buckles) should provide additional insight into how sensitive specific muscle quantities may be to muscle–tendon dynamics and optimization approach.

Our study shows how combining detailed musculoskeletal models with optimization techniques can provide a rich and varied dataset that complements and enhances existing empirical methods used in comparative biomechanics research. Similar to reductionist models [69,70], these models are well suited to theoretical studies that can elucidate underlying principles and constraints governing motion. For example, this study has generated estimates of muscle excitation, force and musculotendon work during walking and running in an ostrich, which were used to identify muscle functional roles. Muscle roles were found to be insensitive to optimization approach, with the bi-articular hip and knee muscles acting as motors and digital flexors acting as springs during stance. The IC and AMB2 were the main drivers of the swing motion. Passive tissues at the hip also appear to play an important role in ostrich running, acting as a strut to prevent excessive hip abduction. Future models should incorporate non-muscular soft tissues and bony stops, which also deserve careful consideration when modelling or performing dynamic analyses of locomotion of fossil taxa.

## Supplementary Material

Supplementary Figures and Tables

## Supplementary Material

Manuscript with Tracked Changes

## Supplementary Material

Response

## Data accessibility

The musculoskeletal model and motion data used in this study are available via Dryad at http://dx.doi.org/10.5061/dryad.fh3h6.
