# Supplementary material for: Inferring muscle functional roles of the ostrich pelvic limb during walking and running using computer optimization
Source: J R Soc Interface. 2016 May;13(118):20160035. doi: 10.1098/rsif.2016.0035 (PMC4892259; doi:10.1098/rsif.2016.0035)

Supplementary Information – Tables

Table S1 – Root mean square (RMS) and peak differences between the experimental and simulated joint angles (degrees) obtained with computed muscle control in walking and running over the entire movement.

Joint Angle (Degrees)	W_{CMCR}		W_{CMCC}		R_{CMCR}		R_{CMCC}	
	RMS Error	Peak Error	RMS Error	Peak Error	RMS Error	Peak Error	RMS Error	Peak Error
**Hip Flexion-Extension**	0.002	0.061	0.001	0.039	0.007	0.148	0.002	0.044
**Hip Ad-Abduction**	0.002	0.106	0.001	0.034	0.005	0.164	0.002	0.065
**Hip Medial-Lateral Rotation**	0.005	0.139	0.003	0.076	0.012	0.347	0.003	0.054
**Knee Flexion-Extension**	0.006	0.201	0.002	0.047	0.024	0.497	0.002	0.069
**Knee Ad-Abduction**	0.005	0.130	0.002	0.103	0.014	0.361	0.004	0.071
**Knee Medial-Lateral Rotatior**	0.005	0.236	0.003	0.071	0.010	0.264	0.005	0.122
**Ankle Flexion-Extension**	0.009	0.325	0.001	0.053	0.051	1.304	0.004	0.093
**MTP Flexion-Extension**	0.010	0.292	0.007	0.313	0.051	1.543	0.008	0.193

Table S2 –Percent of muscle activity (as predicted by the simulations) that occurs during the swing phase. Predictions were calculated by normalizing the entire gait cycle to 100% and then integrating the entire cycle and swing-phase-only portions of the simulated muscle activation patterns.

Simulation	Percent of Muscle Activity in Swing
W_{SO}	**13.9**
W_{CMCR}	**15.6**
W_{CMCC}	**29.8**
R_{SO}	**24.4**
R_{CMCR}	**31.8**
R_{CMCC}	**38.6**

Supplementary Information – Figures

Figure S1 – Example fibre excursions of the digital flexor muscles during ostrich running. Due to having short muscle fibres, these muscles undergo extensive fibre lengthening relative to their optimal fibre length during swing. As a result, passive muscle forces generate a large ankle extension moment that cannot be counteracted by the ankle flexors (TCf,TCt, EDL) alone and a reserve torque is required. Shaded area represents stance and the arrow indicates the point of peak ankle reserve actuator torque.

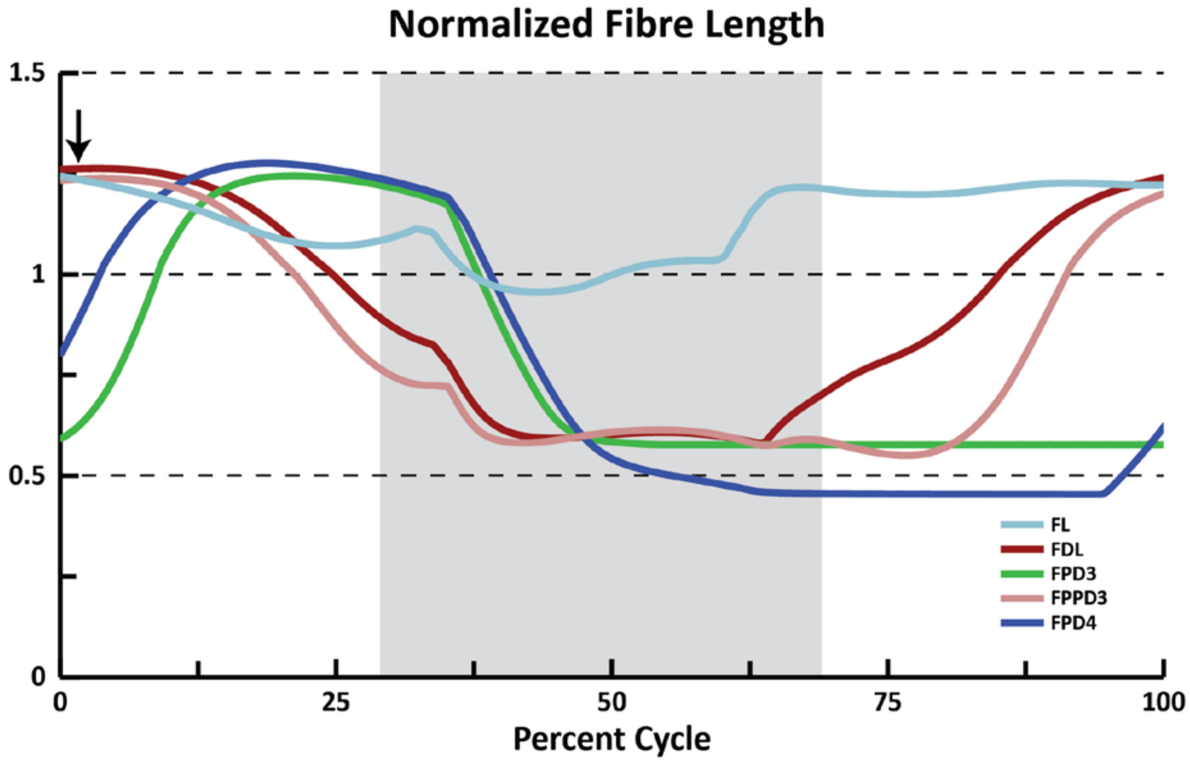

Supplement: Supplementary Figures and Tables [file rsif20160035supp1.pdf]
