# Supplementary material for: Inferring muscle functional roles of the ostrich pelvic limb during walking and running using computer optimization
Source: J R Soc Interface. 2016 May;13(118):20160035. doi: 10.1098/rsif.2016.0035 (PMC4892259; doi:10.1098/rsif.2016.0035)
Supplement: Response [file rsif20160035supp3.doc]

Response to reviewers of:

**Inferring muscle functional roles of the ostrich pelvic limb during walking and running using computer optimization**

Jeffery W. Rankin^1^, Jonas Rubenson^2,3^, John R. Hutchinson^1^InfeIn

^1^Structure and Motion Laboratory, Department of Comparative Biomedical Sciences, The Royal Veterinary College, Hawkshead Lane, Hatfield, Herts, UK

^2^Department of Kinesiology, Pennsylvania State University, University Park, PA, USA

^3^School of Sport Science, Exercise and Health, The University of Western Australia, Perth, WA, Australia

We thank both reviewers for their willingness to continue to review our manuscript. We provide a point-by-point response to the few remaining reviewer’s comments below.

**Referee: 1**

Comments to the Author:

(1) The text added on MTU work (Page 12, Line 57) looks like it may be missing units; should this say, "e.g. ­66.5 vs. ­37.8 J and 49.6 vs. 44.0 J" ?

*Thank you for correcting this oversight. The data are in Joules and we have now added units to the text (J), as suggested.*

(2) I agree with Reviewer 2 that the message of the paper regarding muscle function seems a little muddled, even with the revisions made. Some suggestions that may help clear this up:­

- The term "division of labour" is used several times to broadly describe the results. This may just be semantics and I'm not sure if this is standard terminology in comparative work, but I find its usage here somewhat incompatible with the data this division was inferred from (can you really infer "labour" from EMG or muscle excitation timing?) and potentially misleading, given that the authors later suggest this scheme may be flawed, so perhaps consider removing this term. For example in the abstract, it could be said "Consistent with EMG studies, muscles tended to be excited during only stance or swing..." rather than "Consistent with EMG studies, a clear division of labour existed, with muscles tending..." ­

- Similarly, on Page 13, Line 32, rather than using "division of labour between anatomical groups", could you instead make a statement like "It is common to infer muscle function from muscle activity timing between stance and swing [citations]. The present results suggest this scheme may be flawed or limited because (reasons)."?

*We agree that the term “division of labour” is unnecessary and have removed this phrase from the manuscript, as suggested.*

*In the Abstract, the original sentence “*Consistent with previous avian EMG studies, a clear division of labour was observed in muscle excitation patterns, with muscles tending to be excited during only stance or swing*”* *now reads “*Consistent with previous avian EMG studies, predicted excitation patterns showed that individual muscles tended to be excited primarily during only stance or swing.*”*

*In the discussion the sentence (Page 13, Line 10) “*In both gaits, individual muscle excitation timing and integrated muscle activity occurred primarily during either stance or swing, suggesting a division of labour in which primary muscle functional roles are associated with gait phases (Figures 3-7)*” has been replaced with “*In both gaits, individual muscle excitation timing and integrated muscle activity occurred primarily during either stance or swing, suggesting that primary muscle functional roles may be associated with gait phases (Figures 3-7).”

*The following paragraph (Page 13, Lines 25-49) has been revised (changes underlined) to remove the phrase “division of labour” and improve clarity, which now reads: “*Although a broad division based on gait phases could be identified for individual muscles, this division did not scale to anatomical groups. For example, within the femorotibialis muscle group, FMTM and FMTL were classified as stance phase muscles but FMTIM was classified as a swing phase muscle based on excitation timing. Similarly, the cranial portions of M. iliofibularis (ILFBa) and M. iliotibialis lateralis (ILa) had different classifications from the caudal portions (ILFBp, ILp) during running (Table 4). Previous EMG studies have also suggested that muscles within anatomical groups are differentially excited. Marsh et al. [22] showed that the Mm. femorotibialis and M. iliofibularis usually had two excitation periods during running – one during stance and a second during swing. Gatesy [23] also found the cranial and caudal compartments of the M. iliotibialis lateralis to have distinct activity patterns. Our study, combined with the previous EMG work, highlights the need to exercise caution when assuming that anatomically similar muscles also have similar functions during movement. In addition, the present results further suggest that even general classification of muscles based solely on excitation relative to either stance or swing phase mechanics may be too simplistic. For example, despite their primary activity being clearly associated with either stance or swing, many limb muscles in this study also had small amounts of excitation over transition regions (e.g., late stance to early swing) [22,43]. The reasons for this low level excitation are less clear: activity may be associated with a secondary minor functional role or may be a result of time delays between muscle activity and force generation – future work directed at resolving this uncertainty (e.g., combining simulations with induced acceleration and/or segment power analyses [55-57]) is warranted.”

- When it is suggested further work is needed on clarifying functional roles of muscles (Page 13, Line 48), consider elaborating briefly (but more specifically) on how this could be done, e.g. are the authors suggesting an induced acceleration analysis / muscle contributions to "gait subtasks" (e.g. Neptune et al., 2001)?

*We did intend to suggest combining simulations with analyses specific to understanding how muscles contribute to individual segment movement and energy flow during the movement (e.g., induced acceleration analysis/segment power analysis). We have now explicitly suggested these analyses as avenues for future work and provide references, as suggested by the reviewer (see last sentence of paragraph provided above).*

**Referee: 2**

Comments to the Author:

The authors have done an excellent job of revising and improving their manuscript in response to the feedback provided by the reviewers. I have no further comments or questions.

*We thank the reviewer for his/her assistance in reviewing our manuscript.*
